# Should All Fractions of the Boar Ejaculate Be Prepared for Insemination Rather Than Using the Sperm Rich Only?

**DOI:** 10.3390/biology11020210

**Published:** 2022-01-28

**Authors:** Chiara Luongo, Pedro José Llamas-López, Iván Hernández-Caravaca, Carmen Matás, Francisco Alberto García-Vázquez

**Affiliations:** 1 Departamento de Fisiología, Facultad de Veterinaria, Campus de Excelencia Mare Nostrum Universidad de Murcia, 30100 Murcia, Spain; chiara.luongo@um.es (C.L.); cmatas@um.es (C.M.); 2 Departamento de Tecnología Agroalimentaria, Universidad Miguel Hernández, 03202 Elche, Spain; pedrojosellamas@outlook.es; 3 Departamento de Enfermería Comunitaria, Medicina Preventiva y Salud Pública e Historia de la Ciencia, Facultad Ciencias de la Salud, Campus de Sant Vicent del Raspeig, E-03080 Sant Vicent del Raspeig, Spain; ivan.hernandez@ua.es; 4 Institute for Biomedical Research of Murcia (IMIB-Arrixaca), 30120 Murcia, Spain

**Keywords:** porcine, sperm conservation, ejaculate portions, sperm function, reproduction, litter performance

## Abstract

**Simple Summary:**

The swine industry is constantly looking for efficiency improvement, especially focusing on the artificial insemination (AI) process. One of the trends in AI centers is to maximize the number of doses obtained from one ejaculate. Seminal doses are usually prepared with the sperm-rich fraction or the whole ejaculate, but further studies are needed to understand how to prepare them properly. Thus, this study aims to analyze how accumulative ejaculate fractions may influence sperm storage, AI performance, and offspring. The results indicate that the presence of all ejaculate fractions within seminal doses does not affect either sperm quality or AI performance and offspring health. Therefore, this study highlights the possibility to use the bulk ejaculate for seminal dose preparation, leading to successful AI. Additionally, it results in a more time-efficient preparation of a greater number of seminal doses providing an economic advantage.

**Abstract:**

Boar ejaculate is released in several well-characterized fractions, differing in terms of sperm concentration, seminal plasma volume, and composition. However, the inclusion of the last part of the ejaculate for artificial insemination (AI) purposes is still under debate due to its controversial effects. Thus, there is a need to study the potential synergistic impact of the different ejaculate fractions. We aimed to evaluate the effect of accumulative ejaculate fractions on sperm conservation, AI performance, and offspring health. Ejaculates (*n* = 51) were collected and distributed as follows: F1: sperm-rich fraction; F2: sperm-rich + intermediate fractions; F3: sperm-rich + intermediate + poor fractions. Each group was diluted in a commercial extender, packaged in seminal doses (2000 × 10^6^ sperm/60 mL), and stored at ~16 °C. On day 3 of conservation, sperm were analyzed and used for AI (*n* = 174). High sperm quality was observed after storage without a significant difference between the groups (*p* > 0.05). Moreover, no differences were obtained for AI performance (pregnancy and farrowing rates, and litter size; *p* > 0.05) and offspring health (growth and blood analysis; *p* > 0.05). Conclusively, the presence of all ejaculate fractions within the seminal doses does not impair the reproductive performance, reporting important economic savings according to the economic model included here.

## 1. Introduction

The porcine reproduction industry uses artificial insemination (AI) as the method to achieve fertilization. This fact implies the preparation of seminal doses from ejaculates of selected boars, which involves different steps, such as ejaculate collection, semen dilution, sperm quality control, packaging, distribution, and storage [1]. Today, modern breeding requires semen production in AI specialized centers, which improves the efficiency and accuracy of swine reproduction.

Boar ejaculate is characterized for its high volume (250–300 mL; [2]) and pulsatile ejaculation in well-differentiated fractions. The pre-sperm fraction is discarded because of a high degree of cell debris, urine, and smegma contamination. Then, the sperm-rich fraction is emitted, containing most of the spermatozoa of the ejaculates, which is well recognized by its creamy-white color. Then, the poor fraction is characterized by a lower number of sperm and high content of seminal plasma (watery aspect) [1,3]. Between the rich and poor fraction, there is a transition phase called the intermediate fraction in which the aspect is of a grayish color. Moreover, a gel fraction (tapioca) is expelled progressively during the intermediate fraction and is always discarded from ejaculate collection.

After obtaining the ejaculate, the process of preparing the seminal doses starts. There is a certain controversy in the way of preparing AI doses. Various ways of dilution rate/sperm concentration [4], semen management [5,6,7], semen conservation [8], or inclusion of seminal plasma [9] have been proposed. The rich fraction of the ejaculate is the base foundation of AI dose preparation. However, currently, boar studs are including semi-automatic ejaculate collectors [10,11,12] instead of the traditional manual gloved-hand method, where the entire ejaculate is collected. It is known that boar seminal plasma composition varies depending on the fraction [2,13,14], which influences sperm conservation. However, other factors in addition to seminal plasma composition could impact semen conservation, such as seminal plasma proportion [8,15] or sperm concentration [16,17]. These circumstances with the ongoing trend towards prolonged storage times and lower sperm numbers per semen dose [18,19] open, once again, the controversy of the detrimental effect of seminal plasma inclusion on semen conservation and further fertilization rates [8]. Moreover, the success of post-cervical AI under farm conditions triggered an increased number of insemination doses produced per male and, as a consequence, a reduction in the number of boars needed on the boar stud [20], which elevates the importance of maximizing the efficiency of each ejaculate.

When semen (comprising seminal plasma and spermatozoa) is deposited into the female genital tract, not only does a mere transport of the sperm towards the oocyte start, but an active response is also elicited, including interactions with cells (sperm, oocyte, epithelial cells, leukocytes, or dendritic cells), organs (female genital tract), and fluids (seminal plasma, uterine, and oviductal fluids) (reviewed by [21,22]). These responses and interactions induce changes in the female genital tract [21,23,24,25,26] but also have an impact on embryo development, gene expression [27,28], as well as offspring growth and metabolism [27]. Given the relevance of seminal plasma exposure on offspring in other species, it will be important to study the contributions of seminal plasma in the offspring of porcine species.

The present study aims to evaluate the effect of accumulative fractions of the ejaculate in seminal doses on in vitro sperm quality during conservation, in vivo reproductive performance after AI (fertility and prolificacy) in field conditions, and offspring analysis (growth and blood assay). Moreover, due to the importance of porcine production worldwide, an economic study has been included, taking into account the results of the study.

## 2. Materials and Methods

### 2.1. Ethics

All of the procedures for this study were approved by the Ethical Committee of the University of Murcia on 1 June 2020 (PID2019-106380RB-I00). Through the experiments, animals were handled carefully, avoiding any unnecessary stress. All the experiments were performed in accordance with relevant guidelines and regulations. The study was carried out in compliance with the ARRIVE guidelines (https://arriveguidelines.org/ (accessed on 1 March 2021)).

### 2.2. Boars and Semen Collection

The study was performed from March to September of 2021. A total of 6 fertility-proven boars (Pietrain German Genetics; 30.83 ± 2.63 months of age) showing well-differentiated ejaculate fractions were used for the experiment. Boars were housed in individual pens (according to the European Commission Directive for Pig Welfare) with sawdust in a commercial boar stud (Sergal Gestió Ramadera, Lleida, Spain). Temperature levels were controlled automatically by a climate control system, which maintained the temperature in the room between 18–22 °C. Boars had a restricted feeding regime according to their nutritional requirements. Water was available ad libitum.

A total of 51 ejaculates were collected in a pre-warmed thermal cup using the gloved-hand method. The type of ejaculate depending on the fraction/s included: (1) ejaculate containing only the rich fraction (one fraction—F1) (*n* = 17); (2) ejaculate containing rich fraction (F1) + intermediate fraction (two fractions—F2) (*n* = 17); (3) ejaculate containing rich fraction + intermediate fraction + poor fraction (three fractions—F3) (*n* = 17). The distribution of the ejaculate type and the number of males is shown in Table S1. For each extraction, different types of the ejaculate were collected, distinguished by sight: (F1) composed of the sperm-rich fraction of the ejaculate, characterized by a dense white color. The collection in this case ended with the transition to a less dense white color; (F2) included F1 and the transition fraction between rich and poor fractions, which consisted of a less dense white color of the ejaculate; (F3) included F2 and the poor fraction, characterized by a water-like liquid aspect. In any case, the pre-sperm phase of the ejaculate was discarded, and the gel fraction was removed using a filter. During the trial, semen collection was always carried out by the same technician. The following characteristics of the ejaculates were recorded: volume (mL), number of sperm (×10^6^) per mL, number of sperm (×10^9^) per ejaculate, and number of seminal doses per ejaculate (2000 × 10^6^ sperm/60 mL). Sperm concentration was calculated using an automatic sperm analyzer (Androvision^®^ Minitüb, Tiefenbach, Germany). Moreover, an estimation of the percentage of seminal plasma per dose was performed ((volume of ejaculate/n° of seminal doses per ejaculate) × 100/60 mL (volume of seminal dose)).

### 2.3. Seminal Dose Preparation and Conservation

**Figure 1:**
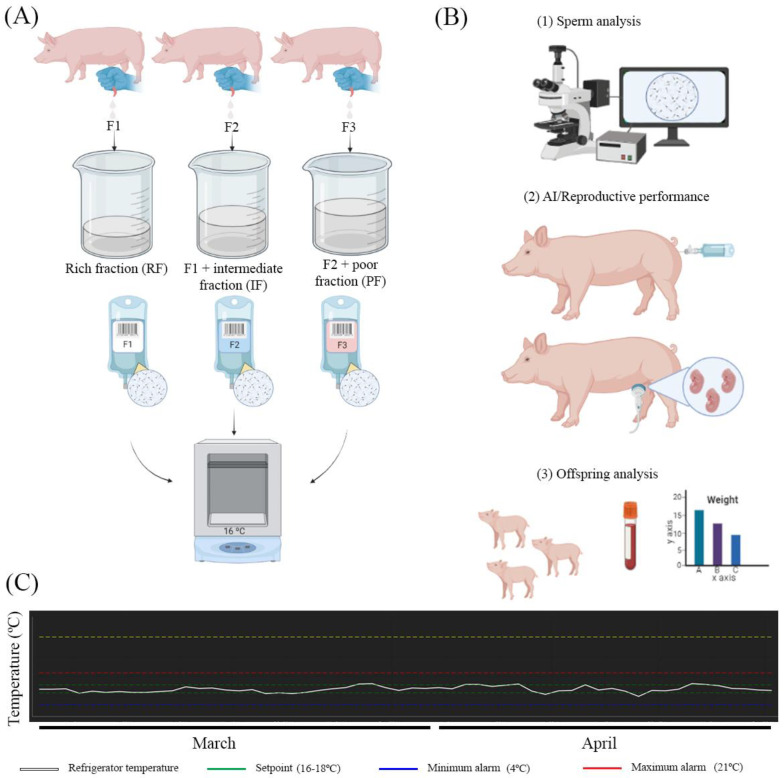
Scheme of the procedures carried out during the study. (**A**) The collection of the semen was performed according to the fractions of the ejaculate (F1 = rich fraction; F2 = F1 + intermediate fraction; F3 = F2 + poor fraction). The seminal doses were prepared for each type of ejaculate and kept at refrigeration until use. (**B**) On day 3 of conservation, the seminal doses were used for controlling the semen quality and for AI. The inseminated sows were diagnosed in pregnancy by ultrasound, and offspring was evaluated (growth and blood analysis). (**C**) Seminal doses were conserved in a temperature-controlled refrigerator during the whole period of the trial (March and April 2021). Images (**A**,**B**) were created on Biorender.com (accessed on 1 March 2021).

Semen samples were diluted in AndroStar^®^ Plus extender (Minitüb, Tiefenbach, Germany) until reaching a final concentration of ~33 × 10^6^ sperm/mL. Semen was packaged in plastic bags (2000 × 10^6^ sperm/60 mL) and color-labeled depending on the type of seminal doses for a better identification at the lab and farm (F1 was a white-colored label; F2 was a blue color; F3 was a pink color) (Figure 1A). Semen preparation was carried out by the same technician during the whole period of the trial. Finally, seminal doses were kept refrigerated until semen evaluation and AI (Figure 1A,B). The temperature inside the refrigerator was monitored (AKO group, Barcelona, Spain) every 15 min during the execution of the trial (from 1 March 2021 to 24 April 2021) (Figure 1C). The average temperature of the conservation was 16.72 ± 0.72 °C (mean ± SD).

### 2.4. Sperm Analysis

Sperm quality from seminal doses (the same doses used in the AI trial) was evaluated at day 3 of storage.

#### 2.4.1. Motility Analysis by CASA

Spermatozoa motility and kinetic parameters were analyzed by the Computer Assisted Semen Analysis (CASA) by ISAS^®^ software (PROiSER R+D S.L., Valencia, Spain) coupled to phase-contrast microscopy (negative-pH 10× objective; Leica DMR, Wetzlar, Germany) and a digital camera (Basler Vision, Ahrensburg, Germany). Each sample was warmed at 38 °C (heat block CH100, Biosan Laboratories, Inc., Warren, MI, USA) for 10 min before evaluation. Then, a 4 µL drop of the sample was placed in a prewarmed (38 °C) chamber (20 micron Spermtrack^®^ chamber, Proiser R+D, SL; Paterna, Spain), and at least 3 fields per sample were recorded. CASA setting parameters used were 25 frames per second and particle size area between 10 and 80 mm^2^. Spermatozoa were considered to be motile when there was an average path velocity (VAP) > 10 μm/s. Progressive motility was considered to exist when there was a straightness (STR) > 45%. The variables analyzed were total motility (%), progressive motility (%), curvilinear velocity (VCL, μm/s), average path velocity (VAP, μm/s), straight line velocity (VSL, μm/s), amplitude of lateral head displacement (ALH, μm), percentage linearity (LIN, ratio of VSL/VCL, %), percentage straightness (STR, ratio of VSL/VAP, %), percentage oscillation (WOB, %), and beat-cross frequency (BCF, Hz).

#### 2.4.2. Viability Assay

For spermatozoa viability, the staining solution was prepared with 50 µL of propidium iodide 500 µg/mL (P-4170 Sigma-Aldrich^®^, Madrid, Spain) in 10 mL of PBS without calcium and magnesium (Sigma-Aldrich^®^, Madrid, Spain). Spermatozoa samples were incubated with propidium iodide solution for 10 min at room temperature in the dark. For the evaluation, spermatozoa were observed in transmitted light brightfield and fluorescence microscopy (Leica^®^ DM4000 Led, Wetzlar, Germany, 495/520 nm) and were classified as live (without fluorescence) or dead (red fluorescence). At least 200 cells per sample were counted.

#### 2.4.3. Acrosome Status

For acrosome status, the staining solution was prepared with 100 µL of Arachis hypogea lectin PNA-FITC 200 µg/mL (Sigma-Aldrich^®^, Madrid, Spain) in 10 mL of PBS without calcium and magnesium. Spermatozoa samples were incubated with PNA-FITC solution for 10 min at room temperature in the dark. For the evaluation, spermatozoa were observed in transmitted light brightfield and fluorescence microscopy (Leica^®^ DM4000 Led, Wetzlar, Germany, 495/520 nm) and were classified as sperm with intact acrosome (without fluorescence) or with damaged acrosome (green fluorescence). At least 200 sperm per sample were counted.

#### 2.4.4. Mitochondrial Activity

For mitochondrial activity, the staining solution was prepared with 10 µL of JC-1 0.017 µg/mL (5,5′,6,6′-tetrachloro-1,1′,3,3′-tetraethylbenzimidazolylcarbocyanine iodide; ThermoFisher Scientific Inc., Waltham, MA, USA) in 10 mL of PBS without calcium and magnesium. Spermatozoa samples were incubated with JC-1 solution for 30 min at 38 °C in the dark. For the evaluation, spermatozoa were observed under fluorescence microscopy (Leica^®^ DM4000 Led, Wetzlar, Germany, 495/520 nm) and were classified as sperm with high mitochondrial membrane potential (orange fluorescence) or sperm with low mitochondrial membrane potential (green fluorescence). At least 200 sperm per sample were counted.

#### 2.4.5. DNA Fragmentation

For DNA fragmentation, a Halomax kit for Sus scrofa (Halotech DNA, Madrid, Spain) was used following the manufacturer’s instructions. Agarose was warmed at 100 °C for 5 min and then transferred to 37 °C for 5 min to equilibrate the temperature. Once agarose reached 37 °C, sperm samples were added to the vials containing agarose (1:2, *v*/*v*) and mixed thoroughly. Then, a 2 µL drop of this suspension was placed onto the slide, covered with a glass coverslip, and left at 4 °C for 10 min to solidify. The coverslip was removed, and the samples were treated with first lysis solution for 5 min and then distilled water for 5 min. Finally, slides were dehydrated with sequential 70 and 100% ethanol and stained with red fluorescent stain (HT-RF S100, Fluored^®^, Halotech DNA, Madrid, Spain). For the evaluation, spermatozoa were observed under fluorescence microscopy (Leica^®^ DM4000 Led, Wetzlar, Germany, 495/520 nm) and were classified as sperm with unfragmented DNA (small and compact halo of chromatin dispersion) or with fragmented DNA (large and spotty halo of chromatin dispersion). At least 200 sperm per sample were counted.

### 2.5. Estrus Detection and Artificial Insemination (AI)

A total of 174 crossbred sows [Large-White X Landrace, Danbred genetic] from a commercial sow farm were used for the study (Genera S.L., Lorca, Spain). At weaning, sows were selected by parity (from 3 to 5; mean parity of 3.74) and body conditions before being randomly assigned to one of the treatment groups (F1, F2, or F3). The body conditions of the sows were assessed at the onset of the estrus visually (BC score 1 to 5; 1 was extremely thin, 5 was extremely fat) and by means of back-fat (BF) and loin depth (LD) measurements. The back-fat and loin thickness of the sows were measured at the P2 point (6.5 cm from the middle line of the last rib) using real-time ultrasound scan equipment with a linear probe (SF1 wireless BF and LD scanner, Sonivet, Beijing, China). A total of 4 measurements per animal were carried out (two on the right side and two on the left side) for the two parameters (BF and LD). After weaning, sows were housed in individual gestation crates with ad libitum access to water and 4.0 kg feed/day until the first AI.

Estrus detection was performed once a day in the presence of a sexually mature boar starting on the day of weaning. The weaning-to-estrus interval (WEI) was 4.28 days on average. AI was performed as previously described [20]. Briefly, sows were inseminated by a post-cervical AI method at estrus onset and every 24 h during the standing reflex period (average of 2.55 ± 0.53 inseminations per animal), using a combined catheter-cannula kit (Soft & Quick^®^, Tecno-Vet, S.L., Barcelona, Spain). Each sow was inseminated by the same boar (seminal doses were stored). The seminal doses were composed of 2000 × 10^6^ sperm in 60 mL. The AI procedure was performed in individual crates. From the first AI until day 25 of gestation, sows were fed daily with 2.0–3.5 kg (depending on the initial BC score) of the same gestation feed. From day 25 until the entry to farrowing, all sows received 2.0 kg/day. From the day of farrowing, sows were fed with 1.0 kg/day, increasing the ration by 1 kg/day until maximum (8 kg/day).

### 2.6. Return to Estrus and Pregnancy Diagnosis

Fertility parameters were monitored with return-to-estrus starting at day 18 after the first insemination by boar exposure, while an experienced worker applied back pressure in search of a standing reflex. Sows showing estrus signs were considered non-pregnant. Moreover, pregnancy was detected by ultrasound 23–28 days after insemination by transabdominal ultrasonography (Echoscan T-300 S, Barcelona, Spain). Pregnant sows were then housed in pens with 8–10 sows/pen.

### 2.7. Farrowing and Litter Performance

Pregnant sows were moved from gestation facilities to the farrowing crates at 110 days of gestation. At farrowing, the following reproductive parameters were recorded: farrowing (%), gestation length (days), the total number of piglets born, and the number of piglets born alive. Moreover, the fecundity index (total number of live piglets born per 100 inseminations) was calculated (farrowing rate multiplied by the number of live piglets born per litter).

### 2.8. Offspring Growth Parameters

Body weight (kg) was evaluated at day 1 and 21 of life (weaning) using a precision scale (ZMISSIL F1-30). Moreover, daily weight gain (DWG, kg) was calculated as follows: weight at day 21—weight at day 1/days from first to second weight measured (21 days).

### 2.9. Blood Collection and Analysis

A total of 81 piglets (41 females and 40 males) from 3 experimental groups were randomly selected and used for blood analysis. Blood samples (about 4–5 mL) were collected (7 days after piglet born) by venipuncture of the jugular vein using a Vacutainer system (BD Vacutainer^®^ 21G 0.8 × 25 mm needle; BD Vacutainer), including lithium heparin tubes. After collection, blood samples were transported to the lab within 1 h in a porexpan box and kept at refrigeration (4 °C) until analysis (within 18 h after collection). The hematological analysis was performed using analyzer equipment (Siemens ADVIA^®^ 120, Holliston, MA, USA), while the biochemical serum parameters were evaluated by the Olympus AU600 and Mindray BS-200E analyzers.

The hematological parameters analyzed were hematocrit (HCT, %), concentration of erythrocytes (RBC, ×10^6^ cells/µL), hemoglobin (HB, g/dL), mean corpuscular volume (MCV, fL), mean corpuscular hemoglobin (MCH, pg), mean corpuscular hemoglobin concentration (MCHC, g/dL), cell hemoglobin concentration mean (CHCM, g/dL), red cell volume (RDW, %), cellular hemoglobin content (CH, pg), cellular hemoglobin distribution width (CHDW, g/dL), hemoglobin concentration distribution width (HDW, g/dL), white blood cells (WBC, ×10^3^ cells/µL), neutrophils (NEU, % and ×10^3^ cells/µL), lymphocytes (LYM, % and ×10^3^ cells/µL), monocytes (MON, % and ×10^3^ cells/µL), eosinophils (EOS, % and ×10^3^ cells/µL), basophils (BAS, % and ×10^3^ cells/µL), platelet indices (platelet (PLT, ×10^3^ cells/µL), platelet crit (PCT, %), mean platelet volume (MPV, fL), platelet distribution width (PDW, %), mean platelet volume component concentration (MPC, g/dL), platelet component distribution width (PCDW, g/dL), mean platelet mass (MPM, pg), platelet mass distribution width (PMDW, pg), and large platelets (large PLT, ×10^3^ cells/µL)) as well as reticulocyte indices (reticulocytes (RET, % and ×10^6^ cells/µL), average size of reticulocytes (MCVr, fL), and average hemoglobin content of reticulocytes (CHr, pg)).

The biochemical serum parameters analyzed were proteins (PRO, g/dL), albumin (ALB, g/dL), globulin (GLO, g/dL), creatinine (CR, mg/dL), urea (URE, mg/dL), glucose (GLU, mg/dL), cholesterol (CHOL, mg/dL), triglycerides (TRI, mg/dL), lipase (LIP, UI/L), creatine kinase (CK, UI/L), alkaline phosphatase (ALP, UI/L), gamma-glutamyl transferase (GGT, UI/L), aspartate aminotransferase (AST, UI/L), alanine aminotransferase (ALT, UI/L), total bilirubin (TBIL, mg/dL), calcium (Ca, mg/dL), potassium (K, mmol/L), sodium (Na, mmol/L), and chlorine (Cl, mmol/L).

### 2.10. Statistical Analysis

Statistical analyses were performed with the SPSS 24.0 software package (IBM SPSS Inc., Chicago, IL, USA). Ejaculate characteristics and semen quality parameters were analyzed for normality by a Kolmogorov–Smirnov test, which showed that all parameters had a normal distribution, except the percentage of seminal plasma per dose. A one-way ANOVA test followed by a post hoc Tukey test was applied. For the variable, which data were not normally distributed, the non-parametric Kruskal–Wallis test was used. Data are represented as the mean ± SD (standard deviation) for ejaculate parameters and means ± SEM (standard error of the mean) for sperm parameters. Differences were considered significant when *p* < 0.05.

For body condition, parity, and weaning-to-estrus interval, the non-parametric Kruskal–Wallis test was used. Regarding BF, LD, number of inseminations, gestation period, total and live-born piglets, fecundity index, weight at day 1 and day 21, and DWG, the assumption of normality was evaluated by a Kolmogorov–Smirnov test. All these variables were not normally distributed, and the non-parametric Kruskal–Wallis test was used. Concerning pregnancy and farrowing rates, a Chi-square test was used for comparison between experimental groups. The results obtained are presented as the mean ± SD, and differences were considered statistically significant when *p* < 0.05.

For blood parameters, the assumption of normality was evaluated by a Kolmogorov–Smirnov test. When normality was fulfilled (RBC, MCH, CHCM, CHDW, WBC, NEU%, LYM, MPC, PCDW, PMDW, RET, MCVr, CHr, PROT, ALB, CR, GLU, CHOL, TRI, AMI, GGT, Ca, K), a one-way ANOVA test followed by a post hoc Tukey test was applied. For those variables which data were not normally distributed, the non-parametric Kruskal–Wallis test was used. The results are represented as the mean ± SD. Values were considered significantly different when *p* < 0.05.

## 3. Results

The results of the ejaculate characteristics are depicted in Table 1, showing significant differences between groups in all of the parameters studied. The number of sperm per mL was significantly higher in the F1 group in comparison with F2 and F3, with F2 being statistically greater than F3. The total number of sperm per ejaculate and the number of seminal doses prepared per each type of ejaculate were statistically greater in F3 than F1, while F2 was similar to both. Having an estimation of six ejaculate collections per boar/month (data provided by a commercial boar stud), the use of F3 had an increase of seminal dose production of 24.16% per month compared to F1, while the use of F2 supposed an increase of 11.18%. After seminal dose preparation, semen was stored for 3 days before in vitro evaluation. Sperm quality did not show significant differences between the experimental groups (Table 2). All the parameters evaluated remained on a high level throughout 3 days of storage (e.g., total motility ranged from 89.11 ± 0.79% to 90.89 ± 0.92%; mitochondrial activity from 91.79 ± 0.34% to 92.37 ± 0.38%).

Inseminated sows showed similar body parameters (BD, BF, LD) in all three experimental groups (Table 3). Moreover, parity and weaning-to-estrus interval (which ranged from 3.72 ± 0.74 to 3.76 ± 0.76 and from 4.21 ± 0.99 to 4.33 ± 0.98 days, respectively) showed no significant differences between the groups (Table 3). A total of 58 sows were inseminated per group, with no significant differences found between them, both in terms of gestation length (which ranged from 115.82 ± 1.09 to 116.02 ± 1.39 days) as well as pregnancy rate and farrowing rate (which ranged from 92.98% to 96.55% and from 82.46% to 89.66%, respectively) (Table 4). Additionally, in analyzing total and live-born piglets, no significant differences were observed, comparing the different types of seminal doses used (Table 4). When the fecundity index was calculated, it was similar between the groups, with a range from 1517.32 ± 466.68 to 1589.60 ± 320.15 piglets born alive. The results concerning the weight of piglets at days 1 and 21 after birth, and DWG are shown in Table 5. The weight at days 1 and 21 did not show significant differences between the groups, as well as the DWG, which ranged from 0.160 ± 0.059 kg to 0.167 ± 0.052 kg. Furthermore, the blood parameters evaluated in the offspring, including hematological and biochemical analyses, were statistically similar between the experimental groups (Tables S2–S5).

Moreover, an economic study was performed, comparing the three experimental groups. This study showed lower costs for seminal doses prepared with F3 (3.18 €) compared to F1 and F2 (3.91 and 3.54 €, respectively) (Table 6). Additionally, F3 showed a seminal dose cost reduction per inseminated sow and piglet born alive compared to F1 and F2 (Table 7).

## 4. Discussion

Nowadays, the preparation of seminal doses is a crucial step in the swine industry since AI is the dominant form of breeding. However, some aspects of its management are still controversial. Previous reports have been focused on the study of the boar ejaculate fractions per separate, claiming a negative impact of the poor fraction inclusion on sperm features. However, the synergy of different boar ejaculate fractions has yet to be elucidated. The present study indicates that under our conditions, the inclusion of the total fractions of the ejaculate in seminal doses does not impair sperm conservation, fertility, prolificacy, nor offspring performance.

Commonly, the rich fraction of the ejaculate, but not the poor fraction, is collected and processed for seminal doses. This process was established because the inclusion of the poor fraction to the seminal doses had a controversial effect during sperm conservation [2,20], suggesting negative impact upon sperm characteristics. However, the incorporation of the semi-automatic ejaculate collection, where the poor fraction is also collected, has again opened this debate. Our results have demonstrated that the inclusion of the total fractions of the ejaculate (rich, intermediate, and poor) did not affect the sperm quality during conservation at 16 °C. With the inclusion of the total ejaculate, the percentage of seminal plasma in the seminal doses increased in comparison with the single use of the rich fraction (~15% vs. ~8%; Table 1). Previous studies have reported a deleterious effect on sperm quality when a high proportion of seminal plasma is present during sperm conservation [9,15]. The inclusion of 10% of bulk seminal plasma reduced the total sperm motility in comparison with a lower level (0.5 and 5%) from the third day of conservation onwards [9]. This result contrasts ours, where a high sperm quality (~90% of total motility) was observed when a high proportion of seminal plasma was present (which ranged from ~8 in F1 to ~15% in F3), although no comparison has been established using lower levels of seminal plasma. Moreover, the sperm concentration used in both studies was different (18 × 10^6^ vs. 30 × 10^6^ sperm/mL), with the diluted semen being more susceptible to the putative adverse effect of seminal plasma [15]. The variations found between the studies are not surprising, considering that many factors can affect the outcome of semen processing and composition. The semen quality after medium–long term storage of sperm should not reduce the fraction of ejaculate or the proportion of seminal plasma used, as there are other factors that play an important role, such as semen processing [29,30] or the type of extender used [8,9,31]. The extender used in our study is recommended for medium-term conservation, which can aid in conserving the sperm quality when different seminal plasma proportions and fraction origin are used. Furthermore, the seminal plasma proteins not only vary between fractions of the ejaculates [13,14], but there are also inter-breed [32] and inter-male variations [33]. In our case, no male effect was observed. It could be that such variations may have been mitigated by the type of extender used, as previously reported by other authors [9], or there is also the chance that the six boars included in the study present a similar performance.

Although no effect on sperm features has been observed during storage using different accumulative ejaculate fractions, the situation in vivo is far from the in vitro analysis. It is known that seminal plasma influences the transition of the sperm through the female genital tract upon deposition [34] and interacts with the female reproductive fluids [22]. Particularly, seminal plasma proteins are involved during these interactions. For instance, AWN spermadhesins and PSP-I/PSP-II heterodimer, mostly abundant in the sperm-rich and poor fraction respectively, adhere to the sperm surface [13]. These proteins are involved during the interaction between sperm and uterine epithelium and fluid, as well as during sperm–zona pellucida binding [22,35]. However, PSPs seem to have a controversial effect, inducing a higher migration of leukocytes within the female reproductive tract [36]. For this reason, the poor fraction is supposed to exert a negative effect on the fertility outcome with respect to the sperm-rich fraction [37]. However, our data showed that the poor fraction combined with other ejaculate fractions (F3) leads to similar fertility outcomes compared with F1 and F2. Thus, seminal plasma volume and composition used seem to have neither a beneficial nor a harmful effect on pregnancy and farrowing rate, probably because this effect may be mitigated by the simultaneous presence of each ejaculate fraction. Nevertheless, there are controversial studies where the fertility rate and the number of embryos were higher in the presence or absence of seminal plasma [17,38]. Based on our findings, they do not mean that the seminal plasma is not acting in some way in sperm modulation during their transit towards the oocyte because the total absence of seminal plasma is not included in our experimental groups. However, they may indicate that seminal plasma present in F1, F2, and F3 is acting similarly for pregnancy and prolificacy. In fact, the infusion of seminal plasma from the entire ejaculate before insemination resulted in a higher number of collected embryos compared to the infusion of phosphate buffer saline as a control [39]. It is known that seminal plasma stimulation of the female tract is not essential for pregnancy success in a pig, but in animals showing poor reproductive performance or less optimal breeding, this fluid can improve reproductive outcomes (reviewed by [21]). Thus, further studies directed to compare F1, F2, and F3 within sows with low reproductive features could elucidate whether the seminal plasma fractions could impact the pregnancy and the number of piglets born.

Likewise, the seminal plasma according to the fractions present in the seminal doses did not influence the offspring’s health. However, evidence suggesting that paternal factors (e.g., seminal plasma) may influence the offspring are emerging [21,27]. This is relevant in assisted reproduction techniques, such as porcine AI, where semen is diluted, reducing the components of this fluid; the addition of extra seminal plasma could improve the outcomes. Although studied in other species, the impact of female responses to seminal plasma in the offspring’s health has not been evaluated in porcine yet. In our case, the type of seminal doses not only differed between them in protein and metabolite composition (as indicated by other studies; [13,14,40]), but also in the percentage of plasma. However, when the piglets born were evaluated for growth and blood analyses, no differences were found between the three experimental groups, indicating a possible lack of influence by the composition and/or percentage of seminal plasma present in the semen doses used. Therefore, we have tested the effect of the seminal plasma depending on the fractions collected from a practical point of view for the swine industry because ejaculates are not devoid of seminal plasma during processing.

Our results indicate that the inclusion of the entire ejaculate in the seminal doses has no detrimental effect in the different stages of swine production, such as sperm conservation, AI, and offspring health. This implies not only benefits from the collection method as mentioned, but also has positive implications on/in the environment and from an economic point of view. At the time of AI, seminal doses are deposited within the female reproductive tract, and a phenomenon consisting of semen loss through the vulva, named backflow, occurs [41,42]. During the semen backflow, part of the extender, which includes antibiotics, reaches the liquid manure [43]. Using the F3 for seminal dose production includes the addition of less extender and, in consequence, fewer antibiotics. Thus, fewer antibiotics will reach the purine through the backflow, helping to reduce the antimicrobial resistance. Antimicrobial resistance is among the most serious public health threats of the 21st century, with a great impact in terms of One Health (reviewed by [44]). In our seminal doses coming from F3, no bacterial growth was observed at day 3 of storage (data not shown). This fact opens the possibility of reducing the amount of antibiotic used because less extender is needed to process these seminal doses. Furthermore, the protein deoxyribonuclease-2-alpha, an acid endonuclease secreted by male accessory glands, is more highly expressed in the poor ejaculate fraction than in previous fractions [13], providing a bactericide activity protecting sperm in the transit along the female genital tract [45]. On the other hand, the economic impact of incorporating the bulk ejaculate (F3) versus other initial fractions (F1 or F2) on seminal doses has been evaluated, considering the results obtained in our study (Table 6 and Table 7). The use of F3 in seminal doses would ensure important savings. Taking into account fixed, variable, and consumable costs (Table 6), the inclusion of F3 leads to a cost reduction compared to F1 and F2. Our analysis reveals that a dose’s cost of F3 could save 0.73 € and 0.36 € in comparison with F1 and F2, respectively. Moreover, obtaining the bulk ejaculate increases the number of sperm collected and, in consequence, the number of doses and therefore the cost of doses processing per collection. This leads to a more efficient use of the boars, increasing the returns on investment, utilization efficiency, and profitability of the system by reducing the boar cost. Even more, the estimation savings per inseminated sow (considering 2.55 AIs per estrus) using F3 compared to F1 are 1.86 € (Table 7). Extrapolating to the piglet born alive (considering the fecundity index obtained for each group) involves a reduced cost of 0.06 € and 0.12 € per piglet for F2 and F3 compared with F1.

## 5. Conclusions

In conclusion, considering all the information reported here, our findings demonstrate that the inclusion of seminal plasma from the entire ejaculate in seminal doses has similar outcomes in terms of sperm conservation, reproductive performance, and piglet development than when only the rich fraction of the ejaculate is used. Moreover, an efficient use of the boar ejaculate increases the return investment, reduces the use of antibiotics, and reduces the cost per insemination and piglet production, which is of great importance for the swine industry.

## Figures and Tables

**Table 1:** Characteristics of the ejaculates from 6 boars used in the experiment. Data are represented as the mean ± SD (standard deviation).. The number of ejaculates evaluated per group (F1, F2, F3) is indicated between brackets.

	F1 (*n* = 17)	F2 (*n* = 17)	F3 (*n* = 17)	*p*-Value
Volume (mL)	102.35 ± 33.12 a	157.35 ± 31.87 b	308.23 ± 126.36 c	<0.0001
N° of Sperm/mL (×10^6^)	540.94 ± 153.68 a	384.58 ± 119.29 b	241.05 ± 96.94 c	<0.0001
N° of Sperm/ejaculate (×10^9^)	52.08 ± 11.20 a	57.90 ± 13.03 ab	64.67 ± 11.19 b	0.012
N° of seminal doses/ejaculate	26.04 ± 5.60 a	28.95 ± 6.51 ab	32.33 ± 5.59 b	0.012
Seminal plasma per dose (%) *	7.91 ± 3.76 a	9.15 ± 2.92 ab	15.19 ± 7.42 b	<0.0001

* The percentage of seminal plasma per dose was estimated as follows: (volume of ejaculate/n° of seminal doses per ejaculate) × 100/60 mL (volume of seminal dose). Different letters (a,b,c) in the same row indicate a significant difference between experimental groups (*p* < 0.05)

**Table 2:** Spermatozoa quality parameters from different accumulative ejaculate fractions (F1, F2, F3) analyzed after 3 days of storage at ~16 °C. Data are expressed as the mean ± SEM (standard error of the mean).

	F1 (*n* = 17)	F2 (*n* = 17)	F3 (*n* = 17)	*p*-Value
Total motility (%)	90.89 ± 0.92	90.32 ± 0.68	89.11 ± 0.79	0.65
Progressive motility (%)	37.53 ± 1.27	35.42 ± 1.89	39.16 ± 1.41	0.61
VCL (µm/s)	61.68 ± 2.72	59.26 ± 2.65	55.00 ± 1.54	0.50
VSL (µm/s)	22.32 ± 0.84	21.53 ± 0.85	23.00 ± 0.73	0.76
VAP (µm/s)	39.32 ± 1.50	38.32 ± 1.06	37.53 ± 0.93	0.82
ALH (µm)	1.95 ± 0.09	2.00 ± 0.06	1.89 ± 0.04	0.82
LIN (%)	38.26 ± 1.69	38.53 ± 1.75	42.53 ± 1.34	0.48
STR (%)	58.16 ± 1.62	57.00 ± 1.83	61.79 ± 1.51	0.47
WOB (%)	64.63 ± 1.25	66.32 ± 1.19	68.68 ± 0.97	0.35
BCF (Hz)	7.26 ± 0.15	6.79 ± 0.14	6.79 ± 0.11	0.24
Viability (%)	93.05 ± 0.25	92.89 ± 0.30	92.11 ± 0.27	0.32
Acrosome integrity (%)	95.16 ± 0.19	94.74 ± 0.23	94.32 ± 0.21	0.27
Mitochondrial activity (%)	92.37 ± 0.34	92.37 ± 0.38	91.79 ± 0.34	0.74
DNA fragmentation (%)	0.42 ± 0.09	1.11 ± 0.28	0.68 ± 0.15	0.33

**Table 3:** Body condition score, back-fat thickness, loin depth, parity, and weaning-to-estrus interval (mean ± standard deviation) in sows from three experimental groups (F1, F2, F3).

Experimental Group	Sows (*n*)	Body Condition Score (1–5)	Back-Fat (mm)	Loin Depth (mm)	Parity	Weaning-to-Estrus Interval (Days)
**F1**	58	2.67 ± 0.66	11.33 ± 3.89	45.19 ± 7.12	3.76 ± 0.76	4.33 ± 0.98
**F2**	58	2.67 ± 0.66	11.24 ± 3.68	45.42 ± 7.78	3.74 ± 0.74	4.21 ± 0.99
**F3**	58	2.62 ± 0.59	11.57 ± 3.98	45.76 ± 7.04	3.72 ± 0.74	4.31 ± 0.90

**Table 4:** Pregnancy rate (%), farrowing rate (%), total litter size, live-born piglets, and fecundity index (mean ± standard deviation) in inseminated sows from three experimental groups (F1, F2, F3).

Experimental Group	Sows (*n*)	Number of Inseminations per Sow	Pregnancy Rate (%)	Farrowing Rate (%)	Total Born Piglets (*n*)	Live-Born Piglets (*n*)	Fecundity Index * (*n*)
**F1**	58	2.50 ± 0.50	92.98	82.46	22.57 ± 4.73	18.45 ± 4.81	1521.12 ± 396.89
**F2**	58	2.62 ± 0.52	96.55	89.66	20.50 ± 6.50	16.92 ± 5.20	1517.32 ± 466.68
**F3**	58	2.53 ± 0.57	96.55	84.48	21.86 ± 4.28	18.82 ± 3.79	1589.60 ± 320.15

* Fecundity index was calculated as follows: farrowing rate multiplied by the number of live-born Piglets per litter.

**Table 5:** Weight at day 1 (kg), weight at day 21 (kg), and daily weight gain (DWG, kg) of piglets derived from inseminated sows from three experimental groups (F1, F2, F3). Data are represented as the mean ± SD (standard deviation).

Experimental Group	Number of Piglets (Male/Female) (*n*)	Weight at Day 1 (kg)	Weight at Day 21 (kg)	DWG * (kg)
**F1**	611 (273/238)	1.363 ± 0.348	4.916 ± 1.200	0.167 ± 0.052
**F2**	672 (299/289)	1.392 ± 0.340	4.792 ± 1.279	0.160 ± 0.059
**F3**	626 (278/282)	1.418 ± 0.349	4.837 ± 1.275	0.162 ± 0.056

* DWG was calculated as follows: weight at day 21—weight at day 1/days from first to second weight measured (21 days).

**Table 6:** Calculation of a seminal dose cost depending on the ejaculate fraction/s included (F1 vs. F2 vs. F3).

	F1	F2	F3
Fixed costs (€) ^1^	3.172	2.853	2.555
Variable costs (€) ^2^	0.315	0.284	0.254
Consumable costs (€) ^3^	0.425	0.403	0.371
Dose packaging (€)	0.096	0.096	0.096
Extender (€)	0.076	0.075	0.067
Osmotized water (€)	0.050	0.049	0.044
PCR (€)	0.203	0.182	0.163
Seminal dose cost (60 mL) (€) ^4^	3.91	3.54	3.18
Costs difference (%)	0.00	−9.53	−20.77

^1^ Fixed costs include the workers’salary, energy, amortization facilities, boars, and others. Fixed costs of a boar per month are estimated at 495 €. Fixed cost per ejaculate fraction was calculated as: number of seminal doses per boar and month/cost of a boar per month (495 €). The number of seminal doses/boar/month was calculated as: n° of seminal doses per ejaculate (data included in Table 2) × 6 (number of collections per month). Data based on a real boar stud. ^2^ Variable costs: feed, medication, and sawdust. Variable costs of a boar per month are estimated at 49.26 €. Variable costs per ejaculate fraction were calculated as: number of seminal doses per boar and month/cost of a boar per month (49.26 €). The number of seminal doses/boar/month was calculated as: n° of seminal doses per ejaculate (data included in Table 2) × 6 (number of collections per month). Data based on a real boar stud. ^3^ Calculated as: dose packaging + extender + osmotized water + PCR (for detection of PRRS virus). ^4^ Calculated as: Fixed costs + variable costs + consumable cost.

**Table 7:** Economic comparison between ejaculate fraction/s included in the seminal dose (F1 vs. F2 vs. F3) in terms of cost reduction per AI and piglet born alive.

	F1	F2	F3
Number of sows inseminated	100	100	100
Number of AI/sow per estrus ^1^	2.55	2.55	2.55
Seminal dose cost (€) ^2^	3.91	3.54	3.18
Seminal dose cost/100 inseminated sows (€) ^3^	997.05	902.70	810.90
Piglets born alive/100 inseminated sows (€) ^4^	1521	1517	1589
Seminal dose cost reduction/100 inseminated sows (€) ^#^	0	94.35 ^†^	186.15 ^††^
Cost reduction/inseminated sow (€) ^#^	0	0.94 ^¥^	1.86 ^¥¥^
Cost reduction/piglet born alive (€) ^#^	0	0.06 *	0.12 **

^1^ Average of AIs per sow performed in our study. ^2^ Calculated in Table 6. ^3^ Calculated as: number of AIs (100) × number of AI/sow (2.55) × seminal dose cost. ^4^ Data collected from our study (Table 4). ^#^ Calculated based on F1 as the seminal dose type reference. ^†^ Calculated as: seminal dose cost per 100 inseminated sows using F1—seminal dose cost per 100 inseminated sows using F3. ^††^ Calculated as: seminal dose cost per 100 inseminated sows using F1—seminal dose cost per 100 inseminated sows using F2. ^¥^ Calculated as: seminal dose cost reduction per 100 inseminated sows using F2/100. ^¥ ¥^ Calculated as: seminal dose cost reduction per 100 inseminated sows using F3/100. * Calculated as: seminal dose cost reduction per 100 inseminated sows using F2/piglets born alive per 100 inseminated sows using F2. ** Calculated as: seminal dose cost reduction per 100 inseminated sows using F3/piglets born alive per 100 inseminated sows using F3.

## Data Availability

All data generated or analyzed during this study are included in the published paper.

## Supplementary Materials

The following are available online at https://www.mdpi.com/article/10.3390/biology11020210/s1, Table S1: Number and type of ejaculates used per male in the study; Table S2: Red blood cells and reticulocyte parameters analyzed in three groups of piglets derived from AI (F1, F2, F3). Data are represented as the mean ± SD (standard deviation); Table S3: White blood cell parameters analyzed in the three groups of piglets derived from AI (F1, F2, F3). Data are represented as the mean ± SD (standard deviation); Table S4: Platelet parameters analyzed in the three groups of piglets derived from AI (F1, F2, F3). Data are represented as the mean ± SD (standard deviation); Table S5: Biochemical serum parameters analyzed in the three groups of piglets derived from AI (F1, F2, F3). Data are represented as the mean ± SD (standard deviation).
