# Supplementary material for: Should All Fractions of the Boar Ejaculate Be Prepared for Insemination Rather Than Using the Sperm Rich Only?
Source: Biology (Basel). 2022 Jan 28;11(2):210. doi: 10.3390/biology11020210 (PMC8869087; doi:10.3390/biology11020210)
Supplement: Supplementary file 1 [file biology-11-00210-s001.zip › biology-1547076-supplementary.pdf]

Table S1. Number and type of ejaculates used per male in the study.

	F1	F2	F3	Total
**Boar 1**	3	3	2	8
**Boar 2**	3	3	3	9
**Boar 3**	2	2	4	9
**Boar 4**	4	3	2	9
**Boar 5**	2	3	3	8
**Boar 6**	3	3	3	9
**Total**	17	17	17	51

Table S2. Red blood cells and reticulocytes parameters analyzed in three groups of piglets derived from AI (F1, F2, F3). Data are represented as mean ± SD (standard deviation).

	F1 (n= 25)	F2 (n= 27)	F3 (n= 29)	p-value
**HTC** (%)	34.85±4.87	33.99±4.13	35.40±3.25	0.437
**RBC** (x10^6 cells/µL)	5.96±0.62	5.77±0.55	5.96±0.45	0.360
**HB** (g/dl)	10.94±1.61	10.71±1.43	11.35±1.15	0.230
**MCV** (fL)	58.32±5.01	58.82±5.02	59.43±3.47	0.666
**MCH** (pg)	18.30±2.01	18.51±1.91	19.03±1.22	0.281
**MCHC** (pg)	31.34±1.27	31.41±1.27	32.04±1.10	0.71
**CHCM** (g/dl)	28.94±1.35	28.87±1.10	29.28±1.23	0.418
**RDW** (%)	18.48±3.78	18.57±3.95	17.07±1.96	0.171
**CH** (pg)	16.94±1.92	17.02±1.64	17.42±1.16	0.493
**CHDW** (g/dl)	3.18±0.39	3.27±0.47	3.08±0.29	0.208
**HDW** (g/dl)	2.38±0.25	2.43±0.35	2.44±0.20	0.659
**RET (%)**	4.33±2.60	4.36±1.71	4.27±1.61	0.985
**RET (**x10^6 cells/µL)	0.25±0.12	0.24±0.08	0.25±0.09	0.986
**McvR** (fL)	52.43±6.84	51.87±6.73	53.28±4.94	0.691
**CHr** (pg)	15.10±1.85	15.17±1.92	15.53±1.39	0.616

Table S3. White blood cells parameters analyzed in the three groups of piglets derived from AI (F1, F2, F3). Data are represented as mean ± SD (standard deviation).

	F1 (n= 25)	F2 (n= 27)	F3 (n= 29)	p-value
**WBC** (%)	9.81±3.13	8.46±2.81	9.26±3.40	0.299

**NEU** (%)	34.20±12.05	31.91±12.40	29.32±8.29	0.269
**NEU (**x10^3 cells/µL)	3.58±2.21	2.89±1.75	2.81±1.51	0.255
**LYM** (%)	55.19±12.66	57.49±13.55	60.94±8.87	0.201
**LYM** (x10^3 cells/µL)	5.18±1.42	4.66±1.47	5.52±1.85	0.145
**MON** (%)	5.78±2.68	6.29±3.08	5.11±2.42	0.273
**MON** (x10^3 cells/µL)	0.58±0.34	0.54±0.34	0.49±0.31	0.635
**EOS** (%)	1.47±1.04	1.26±0.69	1.17±0.62	0.390
**EOS** (x10^3 cells/µL)	0.14±0.10	0.11±0.08	0.11±0.07	0.315
**BAS** (%)	1.20±0.68	1.18±0.63	1.21±0.55	0.982
**BAS** (x10^3 cells/µL)	0.11±0.06	0.09±0.045	0.11±0.09	0.443

Table S4. Platelet parameters analyzed in the three groups of piglets derived from AI (F1, F2, F3). Data are represented as mean ± SD (standard deviation).

	**F1**	**F2**	**F3**	**p-value**
	**(n= 25)**	**(n= 27)**	**(n= 29)**	
**PLT** (x10^3 cells/µL)	249.64±159.19	226.25±146.10	200.06±111.72	0.429
**PCT** (%)	0.34±0.32	0.26±0.17	0.24±0.12	0.236
**MPV** (fL)	12.97±4.10	12.25±3.03	12.65±3.51	0.768
**PDW** (%)	84.00±8.59	80.76±19.52	86.70±14.44	0.338
**MPC** (g/dl)	21.70±2.31	20.72±2.31	20.66±2.32	0.198
**PCDW** (g/dl)	7.19±0.73	7.23±0.95	7.22±0.80	0.983
**MPM** (pg)	1.53±0.19	1.49±0.19	1.43±0.17	0.185
**PMDW** (pg)	0.88±0.13	0.87±0.13	0.84±0.13	0.487
**Large PLT** (x10^3 cells/µL)	51.68±77.39	30.62±32.59	29.20±19.56	0.178

Table S5. Biochemical serum parameters analyzed in the three groups of piglets derived from AI (F1, F2, F3). Data are represented as mean ± SD (standard deviation). Different letters (a, b) in the same row indicate a significant difference between experimental groups ($p < 0.05$).

	**F1**	**F2**	**F3**	**p-value**
	**(n= 25)**	**(n= 27)**	**(n= 29)**	
**PROT** (g/dl)	1.92±0.73	1.55±0.47	2.20±0.66	0.284
**ALB** (g/dl)	3.00±0.33	2.96±0.30	2.92±0.36	0.691
**GLO** (g/dl)	2.21±0.62	2.39±0.36	2.16±0.60	0.262
**CR** (mg/dl)	1.06±0.20	1.00±0.32	1.02±0.16	0.637
**URE** (mg/dl)	19.61±9.8	17.02±6.64	15.76±9.30	0.266
**GLU** (mg/dl)	107.46±15.11	103.94±22.39	104.78±16.74	0.774

**CHOL** (mg/dl)	151.99±42.54	164.67±57.11	156.23±41.87	0.620
**TRI** (mg/dl)	69.40±24.27	77.96±32.04	71.62±20.71	0.467
**LIP** (UI/L)	23.56±13.02	27.10±13.74	20.20±12.74	0.153
**CK** (UI/L)	5298.55±14535.36	4628.94±10123.61	8169.97±17331.45	0.621
**ALP** (UI/L)	1020.19±490.33	1062.45±231.40	1083.40±453.97	0.848
**GGT** (UI/L)	208.38±122.30	241.54±143.57	206.82±84.65	0.480
**AST** (UI/L)	194.16±310.59	154.87±110.76	206.04±269.91	0.721
**ALT** (UI/L)	49.40±40.98	57.05±42.31	60.88±51.39	0.647
**TBIL** (mg/dl)	0.22±0.22	0.20±0.25	0.13±0.15	0.246
**Ca** (mg/dl)	11.41±0.76	11.11±0.51	11.35±0.47	0.159
**K** (mmol/L)	9.20±2.17	9.54±1.31	8.79±1.70	0.291
**Na** (mmol/L)	130.70±11.12	133.66±11.04	129.42±14.68	0.437
**Cl** (mmol/L)	98.19±9.25	100.73±8.57	96.98±12.43	0.391
